# Bladder Injury During Cesarean Delivery

**DOI:** 10.2174/157340480902140102151729

**Published:** 2013-05

**Authors:** Christopher M. Tarney

**Affiliations:** Womack Army Medical Center, Department of Obstetrics and Gynecology, 2817 Reilly Road, Fort Bragg, NC 28307, USA

**Keywords:** Adhesions, bladder injury, cesarean section, cystotomy, repeat cesarean delivery, risk factor, urologic injury.

## Abstract

Cesarean section is the most common surgery performed in the United States with over 30% of deliveries occurring via this route. This number is likely to increase given decreasing rates of vaginal birth after cesarean section (VBAC) and primary cesarean delivery on maternal request, which carries the inherent risk for intraoperative complications. Urologic injury is the most common injury at the time of either obstetric or gynecologic surgery, with the bladder being the most frequent organ damaged. Risk factors for bladder injury during cesarean section include previous cesarean delivery, adhesions, emergent cesarean delivery, and cesarean section performed at the time of the second stage of labor. Fortunately, most bladder injuries are recognized at the time of surgery, which is important, as quick recognition and repair are associated with a significant reduction in patient mortality. Although cesarean delivery is a cornerstone of obstetrics, there is a paucity of data in the literature either supporting or refuting specific techniques that are performed today. There is evidence to support double-layer closure of the hysterotomy, the routine use of adhesive barriers, and performing a Pfannenstiel skin incision versus a vertical midline subumbilical incision to decrease the risk for bladder injury during cesarean section. There is also no evidence that supports the creation of a bladder flap, although routinely performed during cesarean section, as a method to reduce the risk of bladder injury. Finally, more research is needed to determine if indwelling catheterization, exteriorization of the uterus, and methods to extend hysterotomy incision lead to bladder injury.

## BACKGROUND

The etiology of the term “cesarean” is unclear. One myth suggests that Julius Caesar himself was delivered *via* this route, which is unlikely, as his mother was alive after his birth, and during that time surgical delivery was only performed if a mother was dying or deceased. Another possible Latin origin includes the Latin verb *caedere*, meaning to cut. The first written descriptions of a surgery resembling the cesarean section occurred during the Middle Ages [1]. Since this time there have been extensive modifications to the surgical procedure with multiple variations in techniques existing today. Although cesarean section is one of the most important procedures in obstetrics, since the beginning of the twenty-first century there has been limited evidence-based medicine that supports many of the surgical techniques used during the surgery. Young physicians training in the art of obstetrics tend to adopt techniques from senior physicians who perform these techniques based on clinical experience with no evidence-based data to legitimize such approaches.

Cesarean section is the most common surgery performed in the United States. More importantly, in 2009, 1.3 million births (32.3%) in the United States occurred by cesarean section [2]. This number is likely to increase in the future for multiple reasons. First, there is currently an extensive debate over the risks and benefits of performing an elective primary cesarean section on maternal requests, with the American College of Obstetricians and Gynecologists defending the ethics of offering primary section to informed patients [3]. The other concern is that less than 10% of women who have a primary cesarean section will have a successful VBAC, which unfortunately perpetuates the dictum “once a cesarean always a cesarean” [4].

Given the likely increasing rate in cesarean deliveries, obstetricians need to be cognizant of potential complications. Fortunately cesarean delivery has been associated with low rates of maternal morbidity and mortality over the past century. However, the most common complication of pelvic surgery is urologic injury, with bladder injury quoted as the most frequently injured organ during pelvic surgery [5]. The incidence of bladder injury during cesarean section ranges from 0.08 to 0.94% [6-10]. Although surgical injuries to the bladder are infrequent during cesarean section, providers need to be aware of potential complications in order to appropriately counsel patients and also prepare themselves for possible intraoperative complications. Potential ramifications of bladder injury include prolonged operative time, urinary tract infection, prolonged indwelling catheter time, and formation of vesicouterine or vesicovaginal fistula [6, 11-13]. Another important consideration is that prolonged separation from mother and infant contributes to maternal emotional distress, and delayed initiation of breast-feeding has been associated with challenges for mothers desiring to breast-feed. 

The purpose of this review article is to discuss the current literature regarding risk factors for bladder injury during cesarean section and to examine the current evidence evaluating various techniques we can take to mitigate the risk of bladder injury.

## RISK FACTORS

One of the largest studies looking at bladder injury during cesarean section comes from Phipps *et al*. who conducted a case-control study of women undergoing cesarean delivery in which 42 bladder injuries were identified among 14,757 deliveries (incidence 0.28%) [6]. The authors randomly selected two cases from women, who also underwent cesarean delivery, to serve as controls for each of the 42 cases that involved bladder injury. Twenty-eight cases of bladder injury occurred in repeat cesarean deliveries. A simple logistic model suggests women with a prior cesarean delivery are 4.22 times as likely to have a bladder injury at delivery versus those who did not have a previous cesarean delivery [Odds Ratio (OR) 4.22, 95% Confidence Interval (95% CI) 1.79–10.1]. More importantly, adhesions were found in 60% of women who had bladder injury versus 10% of the women among the control group (P < .01). As would be expected, the rate of cystotomy also increases with the increased number of cesarean deliveries: 0.13% first, 0.09% second, 0.28% third, 1.17% fourth, 1.94% fifth, and 4.49% sixth cesarean delivery [14]. 

This study also demonstrated statistically significant differences between cases and controls regarding other risk factors. Bladder injury was more likely to occur during emergent delivery (31% versus 11%), which is similar to other reports [6, 15]. Unfortunately, meticulous and careful dissection is not always the most important priority when attempting to expeditiously deliver a distressed fetus. Bladder injury was also more likely to occur in patients who had cesarean section during labor (83% versus 61%). For patients with a prior cesarean delivery, failed TOLAC (Trial of labor after cesarean section) was seen more in the bladder injury group than control group (64% versus 22%). Concurrent uterine rupture was seen in 14% of bladder injury versus 0% of controls. Finally, 60% of patients with bladder injury were found to have adhesions at the time of repeat cesarean delivery versus the 10% of controls. No statistically significant differences were found regarding type of uterine incision (classical versus low vertical), induction of labor, presence of chorioamnionitis, fetal position, gestational age, or maternal illnesses [6].

The findings of Phipps *et al*. are similar to those published by Rahman *et al*., which was a retrospective study that looked at 7,708 cesarean deliveries in which there were 34 bladder injuries (incidence 0.44%)- 41.2% of bladder injuries occurred in primary cesarean delivery versus 58.5% for repeat cesarean delivery [15]. Similar to Phipps *et al*., Rahman *et al*. demonstrated that the incidence of incidental cystotomy was three times higher in women who had a previous cesarean delivery (0.81% vs. 0.27%, P = .0014) [15]. Most of the patients who had prior cesarean delivery in this group had adhesive disease, with the authors speculating that most bladder injuries occurred secondary to extensive adhesive disease at the lower uterine segment [15]. This study also showed most bladder injuries occurred at the time of opening the peritoneal cavity and during creation of the bladder flap [15].

The primary reason previous cesarean delivery has been found to be a significant risk factor for bladder injury is secondary to adhesive disease formed at the index surgery. The incidence of adhesive disease after a primary cesarean delivery ranges from 46 to 65% [16]. Surgical adhesions can form for approximately one month after surgery, but most adhesion formation will occur immediately at the time of surgery [17, 18]. The pathogenesis of adhesion formation is a complex process in which fibrin, coagulation factors, and inflammatory cells contribute to repairing the damaged peritoneum [17, 19]. Risk factors for the development of adhesions include infection, excessive manipulation of tissue, increased blood loss during surgery, adhesiolysis, tissue ischemia, and infection [16]. 

The timing of cesarean delivery during either the first versus second stage of labor also has an impact on bladder injury. The risk of incidental cystotomy increases when cesarean delivery is performed during the second stage of labor versus the first stage (0.4% versus 0.1%, respectively. P value .004). There are multiple differences between a cesarean section performed during the second versus first stage of labor that contribute to these findings. For women undergoing cesarean delivery for an arrest disorder, specifically arrest of descent, there can be significant surgical trauma around the bladder in attempting to displace and deliver an infant that may be secured in the true pelvis. Furthermore, it is often more difficult to delineate the bladder from the lower uterine segment in a uterus that has been labored. Although incidental cystotomy was found to be higher in patients who were undergoing cesarean delivery during the second stage of labor, this finding was still rare (0.4%) [20]. These findings should not lead one to prematurely counsel a patient toward cesarean delivery in order to mitigate an insignificant complication if the patient has not had an adequate trial of labor.

Finally, the risk of bladder injury in women who had a previous cesarean delivery does not appear to be affected by the planned mode of delivery [21]. This is an important point, as women who are considering a TOLAC require extensive counseling, and physicians must be aware of the risks and benefits of TOLAC. Cahill *et al*. performed a multicenter retrospective study that looked at over 25,000 previous cesarean deliveries in which they calculated that the risk of bladder injury was 0.43%. They determined there was no difference in risk for bladder injury between TOLAC and elective repeat cesarean delivery (0.44% compared with 0.42%) [21]. However, they did determine that the absolute risk of bladder injury in patients with previous cesarean delivery increased as follows: successful VBAC (0.2%), elective repeat cesarean delivery after one previous cesarean (0.3%), elective repeat cesarean delivery after more than one prior cesarean delivery (0.7%), unsuccessful TOLAC (1.1%) [21]. An important point in this is that the data shows unsuccessful TOLAC to be associated with the highest incidence of bladder injury. Regarding composite maternal risk, VBAC is still associated with fewer maternal complications, and an unsuccessful TOLAC is associated with more complications than elective repeat cesarean delivery. Nevertheless, the overall risk of bladder injury is still small at only 1.1%. These results are not significant enough to dissuade women who desire TOLAC. 

## DIAGNOSIS/MANAGEMENT

Ninety-five (95)% of bladder injuries during cesarean section occur at the dome of the bladder with the remaining occurring at the trigone [6]. The average length of bladder injury is 4.2 cm (1–10 cm) [6]. The most likely time bladder injury occurs is during the creation of a bladder flap (43%), another 33% of bladder injuries occur at the time of entry into the peritoneal cavity, and the remaining 24% of the injuries occur during uterine incision or delivery [6]. 

Recognition of bladder injury is imperative in order to take measures during surgery to repair this complication, as inadequate diagnosis and treatment at the time of surgery may lead to grave ramifications. The most important prognostic factor of bladder injury is intraoperative recognition and surgical correction. Injuries repaired intraoperatively have a high likelihood for a return of normal urologic function. However, failure to diagnose a bladder injury during surgery may later lead to vesicovaginal, vesicouterine, or ureterovaginal fistula [22, 23]. Although bladder injury at the time of cesarean section is infrequent, most of the injuries are fortunately identified at the time of surgery - 62% of injuries are identified at the time of delivery of the infant and repair of the hysterotomy [6]. Twenty-one (21)% of bladder injuries are recognized during the creation of the bladder flaps, 12% during entry into the peritoneal cavity, and 5% prior to fascial closure [6]. 

There are multiple intraoperative findings that suggest bladder injury: extravasation of urine, appearance of the Foley bulb, gross hematuria in the Foley bag, and visible detrusor muscle laceration [15]. Multiple techniques are available that can be used to diagnose a bladder injury if one suspects possible injury. The bladder may be instilled with indigo carmine, methylene blue, or sterile milk through a urethral catheter. The extravasation of this material from the bladder enables the surgeon to identify the injury and its location. Surgeons may repeat the instillation of these substances until there is no further leakage of fluid, at which point bladder integrity can be confirmed.

After recognizing an unplanned cystotomy, the first step should be to thoroughly examine the defect to determine the extent of the injury. An important consideration is to determine whether the trigone or ureters have been affected by the cystotomy. As previously demonstrated, most bladder injuries that occur during the time of cesarean occur at the dome of the bladder and are easily repaired with a layered closure. If there is concern whether there may have been ureteral involvement in the injury, then the obstetrician may consider having the anesthesiologist inject 40 mg of Indigo carmine into the patient’s IV to examine for extravasation of dye proximal to the bladder, which would suggest ureteral injury. To reiterate an important point, if there is ever concern for possible ureteral injury that may be out of the scope of practice of the individual surgeon then urology should be consulted intraoperatively.

Various methods have been described on how to perform bladder closure. A simple cystotomy is normally repaired in two to three layers, with the first layer consisting of a simple running closure of the mucosa with a 3–0 absorbable suture [24]. It is important to note here that the use of permanent suture, especially silk, is contraindicated, as it can serve as an impetus for stone formation [25]. The second layer may be closed with a running imbricating stitch using either 2–0 or 3–0 absorbable suture to include the submucosa and muscularis [24]. In order to confirm bladder integrity, one may back fill the bladder with sterile milk or methylene blue dye. Two advantages of using the former material are that it is readily available on labor and delivery, and it does not stain tissue like methylene blue, which may limit one to detect the presence of a recurrent leak [24]. After bladder integrity is confirmed, the surgeon may consider placing a third running stitch of absorbable suture if the serosal margins can be approximated. The bladder should be continuously drained with the use of a Foley catheter for at least 7–10 days postoperatively. Upon removing the Foley catheter, one does not need to obtain a voiding cystourethrogram unless extensive repairs are performed [24].

Overall the febrile morbidity has not been found to be statistically significant in comparing patients who had a bladder injury to those who did not have a bladder injury [26-28]. As a result, there is no evidence at this time to support the use of prophylactic antibiotic therapy for incidental cystotomy. Providers need to individualize their practice based on the clinical scenario but keep into consideration concerns with providing unnecessary antibiotic treatment with regards to facilitating the growth of drug-resistant organisms. Providers may consider obtaining a terminal urinalysis and culture to determine need for antibiotic therapy.

The discussion of ureteral damage and repair is more extensive and outside the scope of this article. However, ureteral injuries occurring proximal to the bladder or within the latter third of the course of the ureter are typically repaired by performing an ureteroneocystostomy. The most important principle of this repair is to ensure there is no tension on the ureter. Surgeons may consider performing a Psoas hitch, which helps mobilize the bladder closer to the side of ureteral injury in order to facilitate a tension-free repair, which is associated with better repair rates.

Although uncommon, incidental cystotomy may be missed at the time of surgery. There are multiple signs and symptoms suggestive of bladder injury that can manifest in the early postoperative period such as hematuria, oliguria, lower abdominal pain, ileus, ascites, peritonitis, sepsis, fistula, and elevation of the blood urea nitrogen/creatinine ration [29]. Retrograde cystography is a useful diagnostic procedure to consider in postoperative patients who are stable and may have potential evidence for urologic injury. Providers may also use the stress cystographic technique, as small injuries may not be identified unless some pressure is placed over the bladder. Abdominal CT with cystography is a valuable tool to use in patients with acute abdominal pain who may also have findings of bladder injury. Finally, one should always consider exploratory laparotomy for patients who are unstable or where there is high suspicion for bladder injury [29].

## PREVENTION

### Adhesions

Adhesions at the time of cesarean delivery are among the most important sources for bladder injury secondary to a distortion of normal anatomy and difficulty dissecting through dense adhesive disease. Surgeons can use various techniques during cesarean section to lessen the chance of creating adhesive disease. Techniques that may reduce adhesions include respect of tissue during dissection, avoidance of increased blood loss, and maintenance of tissue moisture [30, 31]. Furthermore, closure of the hysterotomy and peritoneum may also play a role in adhesion formation.

Hysterotomy incisions are currently closed either in a single- or double-layer closure. Some argue that double-layer closure leads to better hemostasis, but one of the most convincing arguments for double-layer closure is to prevent uterine rupture in a subsequent trial of labor [32, 33]. Double-layer closure is an important consideration when performing a cesarean section on a woman who may be a candidate for a trial of labor in a future pregnancy to prevent her risk for uterine rupture in a subsequent TOLAC. Regarding urologic injury, the choice of closing the hysterotomy with single- versus double-layer may also be a factor in the future prevention of bladder injury. When controlling for confounders, single-layer hysterotomy closure has been found to have a nearly sevenfold increase in the odds of developing bladder adhesions when compared with double-layer closure [34]. 

Double-layer closure likely reduces the exposure of raw surgical surfaces, which can lead to fibrosis and adhesion formation [34]. There are no studies that comment specifically on the type of hysterotomy closure relating to bladder injury. However, one may assume that increasing adhesions proximal to the bladder will inherently increase the risk for bladder injury during subsequent cesarean delivery. As a result, it may be beneficial to perform double-layer closure to decrease the risk of bladder injury. More research is needed to support this claim. 

Peritoneal closure during cesarean section is a controversial topic, as there is conflicting opinion on whether this step decreases the adhesion rate. A Cochrane review examining nine trials demonstrated not closing the peritoneum has been found to show improved short-term benefits such as shorter operative time, decreased postoperative fever, and decreased postoperative hospitalization [35]. Alpay *et al*. showed that there was no difference in adhesive disease if the surgeon closed both the parietal and visceral peritoneum [36]. However, Cheung *et al*. performed a systematic review and metaanalysis that demonstrated that there is a 2.6% increased risk for adhesion formation in patients who did not have closure of the peritoneum versus patients who had closure of the peritoneum (OR 2.6, 95% CI 1.48–4.56) [37]. These findings were similar to those demonstrated by Lyell *et al*., which showed parietal peritoneal closure at primary cesarean delivery was associated with a nearly fivefold decreased risk for adhesions (OR .20, 95% CI .08–.49) [38]. The current conflicting data with no evidence examining the long-term effects of closing the peritoneum provides limited evidence to justify peritoneal closure at this time.

Finally, there are multiple adhesive barriers {Seprafilm (Genzyme Biosurgery, Framingham, MA, USA); Interceed (Ethicon, Johnson & Johnson Company, USA)} on the market today that are theorized to mitigate the formation of adhesions. Multiple studies have demonstrated that adhesive barriers placed at the time of laparotomy are beneficial in reducing the risk of adhesion formation [39, 40]. Additionally, a retrospective cohort study recently identified that the use of adhesive barriers in comparison with no use of adhesive barriers was found to have a significant reduction of adhesions at the time of the next surgery. Two hundred and sixty-two women who had a primary cesarean delivery were followed with 43% of women having repeat cesarean delivery. Of these 112 women, 74% who had an adhesive barrier placed at the initial surgery were found to have no adhesions at the time of repeat cesarean delivery. However, only 22% of women who did not have an adhesive barrier were found to have no adhesions at repeat cesarean delivery (P = 0.011) [41].

### Bladder Flap

There are many variations in surgical technique during cesarean section with one of the most controversial being whether to create a bladder flap. The bladder flap is created by first identifying the vesicouterine peritoneum and then making a horizontal incision just superior to this line to allow the surgeon to push the bladder caudally. Although one may think the initial reason to perform a bladder flap was to prevent urologic injury, in actuality surgeons initially created a bladder flap to prevent spread of intrauterine infection to the peritoneal cavity during the preantibiotic era [42]. A secondary benefit of the bladder flap was then believed to prevent injury to the bladder at time of delivery [42]. However, there is no evidence to support this claim. Hohlagschwandtner *et al*. performed a small randomized clinical trial (n = 102) showing the omission of a bladder flap leads to a reduction in the time from incision to delivery, reduced blood loss, and decreased need for analgesia. This study did not determine whether bladder flap creation has any effect on bladder injury, as the required sample size would have to be over 40,000 to show statistical significance since the rate of bladder injury is so small [43].

Tuuli *et al*. conducted another small, randomized clinical trial (n = 258) that examined the utility of the bladder flap with the primary outcome looking at total operating room time. Secondary outcomes were bladder injury, incision-to-delivery time, incision-to-fascia time, estimated blood loss, postoperative microhematuria, postoperative pain, hospital days, endometritis, and urinary tract infections. They identified that omission of the bladder flap at both primary and repeat cesarean deliveries does not increase intraoperative or postoperative complications. However, this study also did not power their study to demonstrate whether omission of the bladder flap decreased the rate of bladder injury [44]. 

Although at this time there are no studies demonstrating whether creating a bladder flap reduces the incidence of bladder injury, there are theoretical reasons why one could 

argue against bladder flap creation. One of the most convincing arguments against the creation of a bladder flap is that most bladder injuries occur while attempting to create a bladder flap [10, 45]. Also, if the hysterotomy is created just above the vesicouterine peritoneal fold, then the bladder naturally descends from the hysterotomy. Forgoing the creation of a bladder flap also leads to less bleeding and vascular injury. This limits the need for hemostatic sutures, which are often placed in close proximity to the bladder [43]. At this time there is no definitive data to argue for or against bladder flap creation with regards to bladder injury. Unfortunately, a significantly large study would be required to determine statistical significance. Nevertheless, the current data suggests that one may argue against routinely creating a bladder flap during cesarean section unless there is a specific indication for bladder dissection. 

### Urinary Catheter

The placement of a urinary catheter prior to cesarean delivery is another method to reduce risk of bladder injury. A urinary catheter allows the surgeon better visualization of the bladder as he or she is able to palpate the catheter bulb if there is question on the location of the bladder, which may be distorted from previous surgery. Having a continuous catheter at the time of surgery allows for bladder decompression, which can prevent incidental cystotomy at the time of peritoneal entry and during creation of the hysterotomy. Moreover, a distended bladder will likely make surgical exposure more difficult. There is one systematic review looking at the benefit of a urinary catheter during cesarean section. The study demonstrated that nonuse of a urinary catheter is associated with a lower incidence of urinary tract infections, less time until the first void, and less time until ambulation, but there is no increase in intraoperative complications. Unfortunately, this study was not powered to show statistical difference regarding bladder injury. The authors concluded that these findings suggest routine use of indwelling catheters may not be necessary. However, more research is needed on this topic before there is evidence to support discontinuation of indwelling catheters during cesarean section [46].

### Abdominal Incision

The Pfannenstiel incision is probably the most common skin incision used at the time of primary and secondary cesarean delivery. This incision consists of performing a slightly curved horizontal incision approximately two to three centimeters above the pubic symphysis. However, some experts argue that a vertical midline subumbilical incision provides for safer entry into the abdominal cavity in patients who have had multiple abdominal surgeries. The only data regarding this type of abdominal incision and the effects of bladder and/or bowel injury comes from a retrospective analysis of 3164 women undergoing repeat cesarean delivery in which 2713 women had a Pfannenstiel skin incision versus 451 women who had a vertical midline subumbilical skin incision [47]. When comparing Pfannenstiel incision to vertical midline subumbilical incision, this study demonstrated that vertical midline subumbilical incision had a significantly higher risk for bladder injury (P < .0001, OR 6.7, 95% CI 2.6–16.5) [47]. The incidence of bladder injury was 0.33% for women with Pfannenstiel incision versus 2.22% for vertical midline subumbilical incision [47]. 

One could argue one reason for increasing bladder injury with performing a vertical midline subumbilical incision is that surgeons tend to perform this incision on patients with complicated surgical histories, which by itself inherently places them at risk for bladder injury. One of the benefits of this study is the authors performed a multivariate analysis for the effect of confounders such as number of cesarean deliveries, operator experience, and adhesions in which they still maintained a significant association with risk of injury to the bladder (P < .009, OR 3.89, 95% CI 1.4–8.9) [47]. One possible explanation for the increased risk with a vertical midline subumbilical incision is that the inferior end of the incision may be carried over the bladder, which may be adhered higher over the lower uterine segment secondary to previous adhesions [47]. At this time there are no other studies that examine the effects of an abdominal incision, which would suggest that surgeons should consider performing Pfannenstiel incision during cesarean delivery unless the patient has other indications to perform a vertical midline subumbilical incision.

### Uterine Incision

There are various techniques for creating a uterine incision: low horizontal, low vertical, or classical. Complications during delivery of the infant may also require the surgeon to extend a low transverse incision to create a J or T incision. Currently, 90% of uterine incisions are created with a low transverse incision [48]. After performing the incision, there are variations on how to extend the incision to allow enough room to deliver the infant. One can use sharp dissection by using the bandage scissors to extend the incision laterally and superiorly. In contrast, one may also extend the incision through blunt dissection. The first approach requires the surgeon to have his or her back face toward the patient’s head and then use an index and pointer finger to extend the incision laterally and superiorly. The second approach is by placing the surgeon’s pointer and index fingers into the incision and then placing force both cephalad and caudally to extend the incision. Although not specifically described, one should direct more force cephalad as to prevent possible bladder injury inadvertently created by too much force placed caudally.

Rodriguez *et al*. conducted a systematic review with metaanalysis comparing blunt with sharp expansion of a low transverse hysterotomy, which showed no differences in the incidence of extension, estimated blood loss, or ease of delivery; this study did not comment on bladder injury [49]. Cromi *et al*. performed a randomized comparison of 811 patients to determine if there were any differences regarding the incidence of unintended extensions between extending the hysterotomy with blunt dissection using a transversal direction versus a cephalad-caudad direction. This study showed that the incidence of unintended extension was significantly higher in the transversal expansion group compared with the cephalad-caudad group (7.4% versus 3.7%, P = 0.03). However, this study was not able to determine whether the type of incision has any risk for bladder injury, as no bladder injuries were found in either group [50].

At the time of this article, there is no evidence that compares the type of uterine incision and method to extend the incision with regards to risk for bladder injury. Research is needed on this subject to determine whether a specific type of incision or method to extend the incision have any risk for bladder injury.

### Exteriorization of the Uterus

Upon delivery of the infant and placenta, some surgeons will remove the uterus from the abdominal cavity (exteriorization) to allow for better visualization during closure of the hysterotomy. There is conflicting data on the risks and benefits of exteriorizing the uterus. A Cochrane review examining six studies, which compared extra-abdominal from intra-abdominal uterine repair, found exteriorization to be associated with a significant decrease in postoperative fever [51]. Two other large studies demonstrated that there were no differences regarding intraoperative complications when comparing hysterotomy closure to either extra-abdominally or intra-abdominally [52, 53]. The routine exteriorization of the uterus is unlikely to decrease the incidence of bladder injury. Nevertheless, a classical tenet of surgery is to ensure adequate visualization of the surgical field. Therefore, the individual surgeon must determine whether he or she believes exteriorization of the uterus will facilitate a better field of view as to prevent bladder injury. 

## CONCLUSION

The incidence of bladder injury during cesarean section is relatively infrequent. However, the rate of cesarean delivery is high and is expected to rise given decreasing VBAC rates in addition to performing cesarean delivery on maternal request. The most significant risk factor for bladder injury during cesarean section is previous cesarean delivery due to adhesive disease. As a result, providers must recognize and plan for possible complications associated with operating on patients with a history of multiple cesarean deliveries. Unfortunately, there is limited evidence supporting various techniques with regards to decreasing the risk of bladder injury. Although urological injury is a grave fear for surgeons, one should be reassured that bladder injuries detected and repaired intraoperatively are not associated with either short- or long-term complications. 
